# An Outbreak of *Trichophyton quinckeanum* Zoonotic Infections in the Czech Republic Transmitted from Cats and Dogs

**DOI:** 10.3390/jof7090684

**Published:** 2021-08-25

**Authors:** Pavlína Lysková, Radim Dobiáš, Adéla Čmoková, Miroslav Kolařík, Petr Hamal, Kateřina Šmatláková, Jan Hušek, Karel Mencl, Nad’a Mallátová, Zora Poláčková, Anežka Krnáčová, Kristýna Palkovičová, Daniela Jablonská, Jitka Macháčová, Zdeněk Drlík, Denisa Bázsóová, Pavla Jaworská, Lucie Svobodová, Vit Hubka

**Affiliations:** 1 Department of Parasitology, Mycology and Mycobacteriology, Public Health Institute in Ústí nad Labem, 186 00 Prague, Czech Republic; pavlina.lyskova@zuusti.cz; 2 Department of Bacteriology and Mycology, Public Health Institute Ostrava, 702 00 Ostrava, Czech Republic; radim.dobias@seznam.cz (R.D.); denisa.bazsoova@zuova.cz (D.B.); pavla.jaworska@zuova.cz (P.J.); 3 Department of Biomedical Sciences, Institute of Microbiology and Immunology, Faculty of Medicine, University of Ostrava, 703 00 Ostrava, Czech Republic; 4 Department of Botany, Faculty of Science, Charles University, 128 01 Prague, Czech Republic; adela.cmokova@natur.cuni.cz (A.Č.); miroslavkolarik@seznam.cz (M.K.); 5 Laboratory of Fungal Genetics and Metabolism, Institute of Microbiology, Czech Academy of Sciences, 142 20 Prague, Czech Republic; 6 Department of Microbiology, University Hospital Olomouc, 775 15 Olomouc, Czech Republic; luciettasv@seznam.cz; 7 Veterinární Klinika MVDr. Vlastimil Pospíšil, 68 201 Vyškov, Czech Republic; katka.smatlakova@seznam.cz; 8 Veterinární Klinika Veterix, 79 601 Prostějov, Czech Republic; info@veterix.cz; 9 Department of Clinical Microbiology, Pardubice Regional Hospital, 53 203 Pardubice, Czech Republic; mencl.karel@tiscali.cz; 10 Laboratory of Mycology and Parasitology, Hospital České Budějovice, 370 01 České Budějovice, Czech Republic; mallatova@nemcb.cz; 11 Department of Skin and Venereal Diseases, University Hospital Olomouc, 775 15 Olomouc, Czech Republic; zora.polackova@fnol.cz (Z.P.); derma.mohelnice@email.cz (Z.D.); 12 Department of Dermatology, Přerov Hospital, 751 52 Přerov, Czech Republic; krnacova.a@seznam.cz; 13 Dermatology Center DERMI s.r.o., 639 00 Brno, Czech Republic; kristyna@palkovicova.sk; 14 Dermatology Center, Hranice Hospital, 753 22 Hranice, Czech Republic; jablonskadaniela@seznam.cz; 15 Dermatology Center, 757 01 Valašské Meziříčí, Czech Republic; mach.jitka@centrum.cz; 16 Dr. Drlik Dermatovenereology, 789 85 Mohelnice, Czech Republic

**Keywords:** zoophilic dermatophytes, tinea capitis, tinea corporis, antifungal susceptibility testing, EUCAST, terbinafine, zoonotic infections, MALDI-TOF mass spectrometry

## Abstract

*Trichophyton quinckeanum,* a zoophilic dermatophyte mostly known as the causative agent of rodent favus, is relatively rarely reported to cause human infections. Indeed, no infections were detected in Czechia between 2012 and 2015 despite routine verification of species identification by ITS rDNA sequencing. By contrast, 25 human and 11 animal cases of infection were documented from December 2016 to December 2020 and the rates tended to grow every following year. Interestingly, most of the cases were reported in the Olomouc region, suggesting a local outbreak. We bring the evidence that human *T. quinckeanum* infections are most commonly contracted from infected cats or, less frequently, dogs. Although rodents or contaminated soil and environment could be the source of infection to cats and dogs, the occurrence of infections in multiple animals in the same household suggests direct transmission among animals. Confirmation of the identification by molecular methods is highly recommended due to morphological similarity with *T. mentagrophytes/T. interdigitale*. Antifungal susceptibility testing of isolates to eight antifungals was performed using EUCAST methodology (E.Def 11.0). Among the tested antifungals, terbinafine, amorolfine, ciclopirox and efinaconazole were most potent in vitro and elevated minimum inhibitory concentrations were obtained for fluconazole and ketoconazole.

## 1. Introduction

The zoophilic species *Trichophyton quinckeanum* was earlier known as a variety of *T. mentagrophytes*, that is, *T. mentagrophytes* var. *quinckeanum* [1,2]. In the past, the taxonomic status of this pathogen was the subject of many controversies which have been reviewed in detail by Beguin et al. [3]. Based on the recently published taxonomy, which is predominantly based on molecular biology criteria, *T. quinckeanum* again constitutes a distinct species closely related to anthropophilic *T. schoenleinii* [4]. Rodents and camels are considered the main natural reservoirs of *T. quinckeanum* which preferably occurs in the Middle East [5]. In Europe, this pathogen has been only sporadically isolated from humans, and there have been no or only sporadic reports of *T. quinckeanum* in Czechia [1,6,7].

Thanks to a large epidemiological study on dermatophytosis ongoing in Czechia since 2011, exact prospective data substantiated by molecularly identified isolates are available [7,8]. This has led to the detection of an increasing number of cases of infections in recent years which are summarized in this study. In addition to an analysis of epidemiological data, we searched for possible sources of infections and characterized antifungal susceptibility patterns, morphology and MALDI-TOF mass spectrometry (MS) in obtained isolates to offer appropriate treatment options and diagnostic tools.

## 2. Materials and Methods

### 2.1. Dermatophyte Isolates, Specimen Collection and Examination

Dermatophytes from human tinea infections were collected prospectively from eight clinical laboratories across Czechia since 2012 as described previously [7,8]. During this period, more than 15,000 dermatophytosis cases were recorded, representing all clinical types of tinea, with approximately 1500–1700 cases registered per year. Identification of all dermatophytes except *T. rubrum* was confirmed by internal transcribed spacer (ITS) rDNA sequencing (see below). A total of 36 isolates of *T. quinckeanum* were examined in this study. The information on the isolation source of all isolates is provided in Table 1 and Table 2.

Skin lesions were scraped from the edge or entirely (small lesions) using scalpel blades or dermal curette; hairs were plucked and crusts picked from scalp lesions. The collected material was divided for cultivation (Sabouraud’s dextrose agar with cycloheximide and chloramphenicol, and Sabouraud’s dextrose agar without antibiotics, 25 °C) and direct microscopic examination. Skin squames and hairs were mounted between the glass slide and coverslip in a drop of 20% KOH with Parker ink or Blankophor (Bayer, Leverkusen, Germany) [9], depending on the laboratory. Microscopic examination was performed under a light or fluorescence microscope.

### 2.2. Culture, Morphology, Physiology and Preservation

The isolates of *T. quinckeanum* were grown on a set of agar media, including malt extract agar (MEA; Oxoid, Basingstoke, UK), Sabouraud dextrose agar (SDA; HiMedia, Mumbai, India) and potato dextrose agar (PDA; HiMedia) at 25 and 37 °C. Colony color determinations were performed using the ISCC-NBS centroid color charts [10]. Micromorphology was documented as described previously [11].

Physiological tests on T1–T7 agar media were performed according to the procedure described previously [12,13], with commercial (HiMedia) as well as laboratory-made media being tested. The tests included T1 (vitamin-free casamino acid agar); basal medium for agars T2–T5, T2 (T1 enriched with inositol), T3 (T2 enriched with thiamine), T4 (T1 enriched with thiamine) and T5 (T1 enriched with nicotinic acid); physiological test T6 based on vitamin-free ammonium nitrate agar; basal medium for agar T7 (T6 enriched with L-histidine). SGA, MEA and T1–T7 growth at 26 ± 1 °C were assessed after 7 and 14 days of culture.

The isolates were deposited into the Culture Collection of Fungi (CCF), Department of Botany, Charles University, Prague, under accession numbers listed in Table 1 and Table 2.

### 2.3. Molecular Methods

Genomic DNA was extracted from seven-day-old colonies using Fungal/Bacterial Miniprep Kit (Zymo research, Irvine, CA, USA). The quality of the extracted DNA was evaluated with NanoDrop 1000 Spectrophotometer. The ITS rDNA region (ITS1-5.8S-ITS2 cluster) was amplified using the forward primer ITS1F and reverse primers ITS4 or NL4 [14,15]. Reaction volume of 20 µL contained 1 µL (50 ng mL^−1^) of DNA, 0.3 µL of both primers (25 pM mL^−1^), 0.2 µL of My Taq Polymerase and 4 μL of 5 × My Taq PCR buffer (Bioline, London, UK). PCR conditions followed the protocol described by Hubka et al. [16]. PCR product purification followed the previously described protocol [17]. Automated sequencing was performed at Macrogen Sequencing Service (Amsterdam, The Netherlands) using both terminal primers.

### 2.4. MALDI-TOF Mass Spectrometry

Thirty-six isolates of *T. quinckeanum* were analyzed by MALDI-TOF-MS and compared with spectra of other common zoophilic *Trichophyton* species occurring in Czechia, namely the *T. mentagrophytes/T. interdigitale* complex (*n* = 20), *T. benhamiae* var. *luteum* (*n* = 20) and *T. erinacei* (*n* = 20). Culture and protein mass extraction for MALDI analysis was carried out based on recommendations of the manufacturer. Following complete washing and drying of sample pellets, each sample was coated with 1 µL of the matrix (HCCA portioned; Bruker Daltonics GmbH, Bremen, Germany), and again dried at room temperature. MALDI-TOF MS measurements were performed with the Microflex LT mass spectrometer (Bruker Daltonics GmbH, manufacturer). The MALDI Biotyper 3.1 software was used to analyze spectra. FlexAnalysis ver. 3.4 (Bruker Daltonics GmbH) was used for comparison of *T. quinckeanum* mass spectra with other closely related zoophilic dermatophytes. The data generated in the present study were compared with previously published reference spectra from Germany [5].

### 2.5. Antifungal Susceptibility Testing

Fluconazole in a powder form was obtained from Pfizer Pharmaceutical Group (New York, NY, USA); terbinafine, itraconazole, ketoconazole, clotrimazole, efinaconazole, amorolfine and ciclopirox were obtained from Merck (Prague, Czech Republic).

Broth microdilution was performed according to the EUCAST document E.Def 11.0 for dermatophyte testing [18]. Briefly, the isolates were incubated at 25 °C on SDA (Trios, Prague, Czech Republic). Inoculum suspensions were created from one- to two-week-old colonies (to achieve acceptable sporulation). The suspension was prepared in sterile distilled water supplemented with 0.1% Tween 20 and filtered using sterile nylon filters with 11 μm pore size (Merck). The suspension was adjusted to a concentration of 2–5 × 10^6^ conidia per mL by counting the conidia in a hemocytometer chamber. The suspension was then diluted 1:10 with sterile distilled water to obtain the final working inoculum 2–5 × 10^5^ colony-forming units per mL. The inoculum suspension was supplemented with chloramphenicol (Merck; final concentration 50 mg/L) and cycloheximide (Merck; final concentration 300 mg/L). Microplates were incubated at 25 °C in ambient air for 5 days. The endpoints, minimum inhibitory concentrations (MICs), were read visually (the drug concentration yielding no visible growth by eye was scored as the MIC value) and spectrophotometrically (50% inhibition) [18]. *Candida parapsilosis* ATCC 22019 and *C. krusei* ATCC 6258 were used as quality control strains.

MIC ranges, corresponding geometric mean (GM) values, MIC50 (MIC causing inhibition of 50% of the isolates) and MIC90 (MIC causing inhibition of 90% of the isolates) were determined for the antifungal drug and reading method used. For GM calculation, MIC values of ≤0.004 mg/L were set at 0.004 mg/L and MIC values of ≤0.008 mg/L were set at 0.008 mg/L; similarly, MIC values of >64 mg/L were set at 128 mg/L.

## 3. Results

### 3.1. Epidemiological Summary

**Figure 1:**
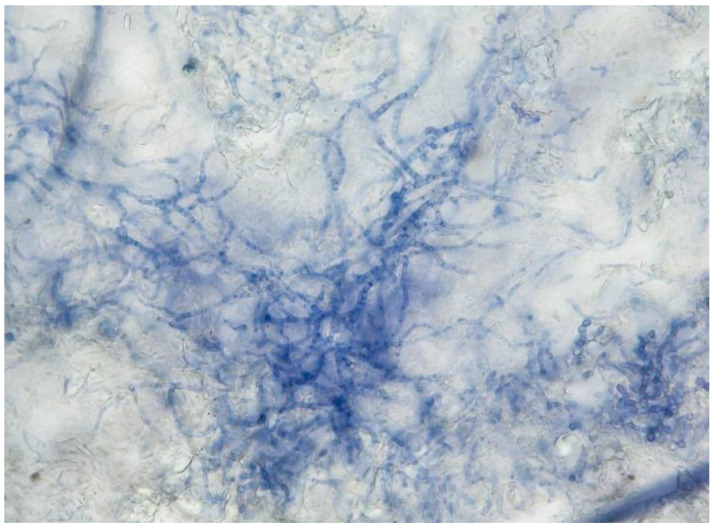
Direct microscopic examination of *Trichophyton quinckeanum* in a skin lesion sample (KOH + Myco-Ink stain, 400× magnification).

**Figure 2:**
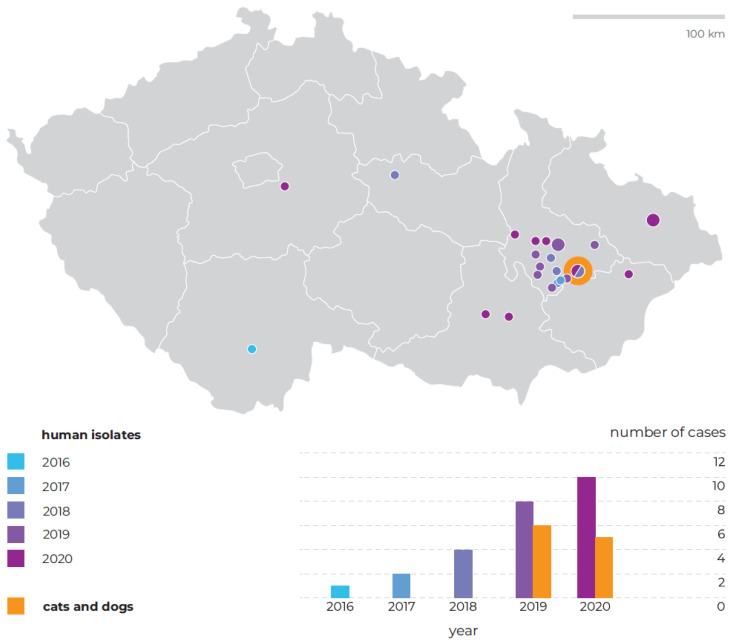
Geographic and temporal distribution of infections with *Trichophyton quinckeanum* in Czechia between 2016 and 2020.

A total of 36 *T. quinckeanum* strains were isolated from Czech human patients (*n* = 25) and animals (*n* = 11; 7 cats and 4 dogs). Microscopic hyphae presented in skin lesion samples are illustrated in Figure 1. All isolates are listed in Table 1 and Table 2. No human cases were detected in Czechia between 2012 and 2015 despite routine identification of dermatophytes by DNA sequencing. The first *T. quinckeanum* strain was isolated in late 2016 and since then, its incidence in Czechia has been noticeably growing (Figure 2). Two human cases were recorded in 2017, four in 2018, eight in 2019, and ten in 2020. Twenty-five strains were isolated in humans and eleven from animals. Almost all cases were detected in the Olomouc region (Figure 2) and predominantly in spring and winter months (Table 1 and Table 2).

*Trichophyton quinckeanum* was more frequently isolated in females (72%). The median age of patients was 23 years (range, 4–77 years); adults predominated (56%) over children and adolescents (≤18 years of age). The infections were mainly manifested as tinea corporis and tinea cruris (*n* = 18; 69.2%), less commonly as tinea capitis (*n* = 4) and tinea faciei (*n* = 4). The patients most commonly reported contacts with cats and dogs in their history (Table 1).

### 3.2. Physiology and Morphology

The strains were unable to grow on T6 basal medium, dissimilar to selected zoophilic species belonging to the *T. benhamiae* complex (*T. benhamiae* var. *luteum*, *T. europeum*, *T. erinacei, T. bullosum* and *T. verrucosum*) [19] and the *T. mentagrophytes/T. interdigitale* complex. The addition of L-histidine to the T6 medium (T7 medium) resulted in a rapid growth of the cultures. Slow-growing zoophilic species, *T. bullosum* and *T. verrucosum*, significantly differed from *T. quinckeanum* in T1, T5 and T7 tests, which were negative. A urease test was positive for all the tested species excluding *T. verrucosum* and *T. bullosum*. Results of physiological tests are summarized in Table 3.

**Figure 3:**
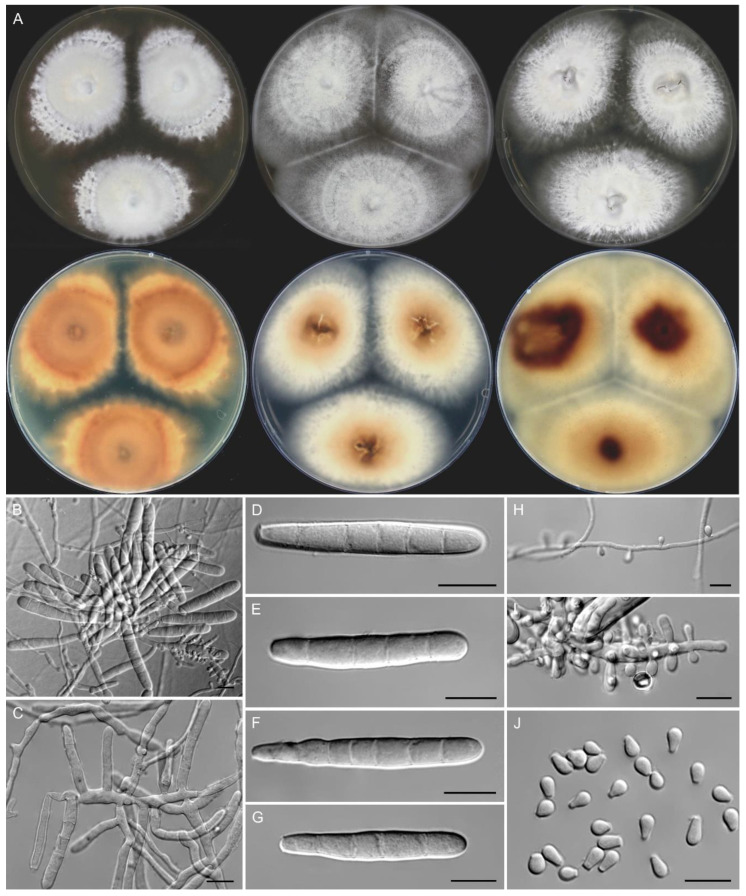
Macro- and micromorphology of *Trichophyton quinckeanum*. The obverse and reverse of colonies after two weeks of culture at 25 °C on Sabouraud dextrose agar, malt extract agar and potato dextrose agar (**A**, from left to right); conidiophores bearing macroconidia (**B**,**C**); free macroconidia (**D**–**G**); conidiophores bearing microconidia (**H**,**I**); free microconidia (**J**). Scale bars = 10 μm.

Macromorphology of *T. quinckeanum* is shown in Figure 3. The growth rates of isolates were comparable to those of *T. mentagrophytes/T. interdigitale* and *T. benhamiae* var. *benhamiae* [19]. The colony diameter ranged from 35 to 47 mm (mean, 39 mm) on SDA, from 28 to 30 mm (mean, 30 mm) on PDA and from 35 to 44 mm (mean, 39 mm) on MEA after 7 days at 25 °C, and from 32 to 35 mm (mean, 33 mm) on SDA after 7 days at 37 °C. Colonies on all media were rapidly spreading, flat with slightly raised, umbonate or cerebriform centers. The colony edges were star-shaped to dendritic on SDA and entire to filiform on MEA and PDA. The colony texture was finely granular to velvety on all media, part of the strains showed a cottony texture, submerged growth, or a mixed pattern with sector growth. The colony color was white (#F2F3F4), yellowish white (#F0EAD6) to dark grayish purple (#50404D); the colony reverse was a vivid orange–yellow (#F6A600), dark purplish red (#673147) to strong yellowish brown (#996515) on SDA, and a pale yellow (#F3E5AB) with strong yellowish brown (#996515) centers on MEA and PDA.

The microconidia were predominantly pyriform to clavate, sized 2.5–5.5 (mean ± SD, 4.1 ± 0.6) × 2–3.5 (2.5 ± 0.2) μm, and borne sessile on conidiophores poorly differentiated from vegetative hyphae or conidiophores branched in a pyramidal fashion with central branches tapering toward the tips. The macroconidia were abundantly produced after 2 weeks of cultivation on MEA in all strains examined micromorphologically (*n* = 10). They were thin-walled, formed at the end of hyphae or poorly differentiated conidiophores, rarely intercalary, cigar-shaped or clavate, 39–57 (48.7 ± 7.4) × 4.5–8 (6.4 ± 0.9) μm, usually consisting of 4–8 cells (2–9 cells; median, 6). Spiral hyphae were present, more abundant in several-week-old cultures.

### 3.3. DNA Sequence-Based Identification

The DNA sequences of the ITS rDNA region were identical in all examined strains of *T. quinckeanum* from human patients as well as from animals. The resulting sequences were deposited into the GenBank database (www.ncbi.nlm.nih.gov) under accession numbers MZ312189–MZ312224.

The sequences were 100% identical to strains associated with recent outbreak of infections in Germany (GenBank accession numbers KY680503, KU257460–KU257462, KU257469) and displayed two and one substitutions in the ITS1 and ITS2 regions, respectively, in comparison with the ex-neotype strain IHEM 13697 (GenBank: MK298745), resulting in a 99.5% similarity. The differentiation of the most closely related species, anthropophilic *T. schoenleinii*, could not be achieved by using ITS region sequencing. Despite the fact that all *T. quinckeanum* isolates showed two substitutions in the ITS1 region compared to the majority of *T. schoenleinii* strains deposited in the GenBank, these substitutions were not conserved for *T. quinckeanum* as a whole. On the other hand, the misidentification of the two species in the clinical laboratory is highly improbable due to their strikingly different morphology and ecology. The differentiation of other dermatophytes by ITS rDNA was possible thanks to many unique substitutions.

### 3.4. MALDI-TOF Mass Spectrometry

**Figure 4:**
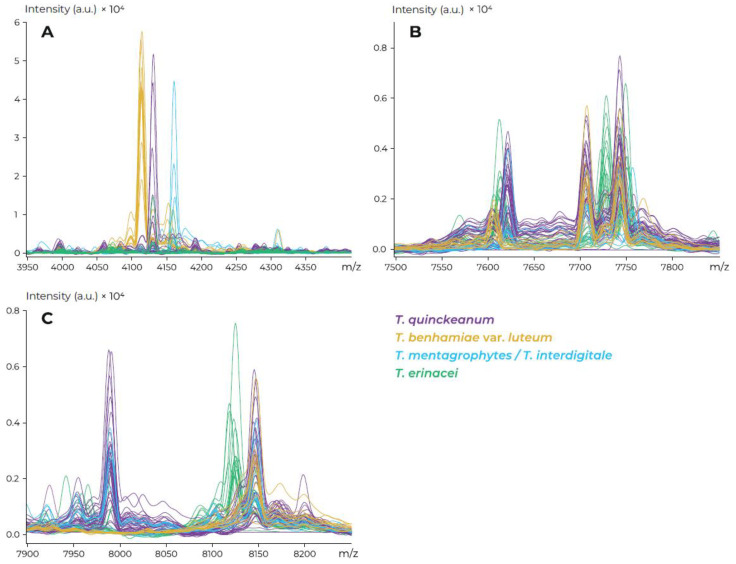
Comparison of mass spectra of *Trichophyton quinckeanum* with common zoophilic *Trichophyton* species, (**A**): 4050–4200; (**B**): 7500–7850; (**C**): 7950–8250.

In the mass range of approximately 4050–4200 *m*/*z*, *T. quinckeanum* could be reliably distinguished from *T. benhamiae* var. *luteum* and the *T. mentagrophytes/T. interdigitale* complex in the specific peaks at 4110 *m*/*z* and 4160 *m*/*z*, respectively (Figure 4A). In the mass range of approximately 7550–8250 *m*/*z*, the MALDI-TOF MS spectra of *T. quinckeanum* were very similar to the above species and their differentiation was not possible (Figure 4B,C).

The differences between *T. erinacei* and *T. quinckeanum* could be found especially in the mass ranges of approximately 7650–7800 *m*/*z* and 8050–8200 *m*/*z* in two specific peaks at 7730 *m*/*z* and 8130–8140 *m*/*z* (Figure 4B,C). Specific peaks in the range of 4110–4160 *m*/*z* could be used in the routine identification of *T. quinckeanum* in practice using the FlexAnalysis tool after the automatic analysis with a MALDI Biotyper.

### 3.5. Antifungal Susceptibility Testing

Results of the susceptibility testing of 36 *T. quinckeanum* strains are given in Table 4. The obtained MICs for fluconazole were elevated in all the tested isolates. MICs for other antifungals tested were low, depending on the reading method used, with generally higher values achieved using visual reading compared to spectrophotometric reading. MICs for amorolfine, ciclopirox and efinaconazole were low. MICs for ketoconazole were elevated. MICs for terbinafine were very low with both testing methods for all isolates, with MIC ≤ 0.016 mg/L.

## 4. Discussion

In Europe, *T. quinckeanum* has been only sporadically isolated from humans. It seemed logical that the pathogen was in decline with an improving standard of life in developed countries as, historically, infections were mostly connected with low social and economic levels and poor hygiene [7,22,23]. For many years, there were no or only rare reports of *T. quinckeanum* in Czechia [1,7,24,25]. The first isolate verified by molecular methods was detected at the end of 2016 and the incidence of cases has been considerably growing since then (Table 1, Figure 2). A similar increase in the incidence of this pathogen was recorded several years earlier in Germany [5].

The species is historically connected with mouse favus or rodent favus in general [1,26], but the occasional hosts are also cats, dogs, rabbits, camels, chickens, horses and sheep [5]. Uhrlaß et al. [5] suspected cats as the main source of human zoonotic infections, which is in agreement with our data. In addition, we showed that dogs can also be the source of infection. It is usually assumed that the infection in domestic animals occurs due to transmission from rodents (mice) and then is eventually transmitted to humans. In this study, we showed that the infection may probably spread among domestic animals independently on rodents as indicated by the occurrence of infection in several animals in the same household (Table 2). This information is valuable because dermatophytes usually spread effectively only in their main host(s) and further transmission among other occasionally infected hosts is rare.

The occurrence of this pathogen in Czechia is a new phenomenon in terms of recent decades and is in contrast to the absence of this species in previous years (2012–2015) during which we confirmed the identification of dermatophytes coming from identical laboratories using ITS rDNA sequencing. Thanks to that, we can confidently exclude the possibility that this pathogen was missed or misidentified due to its morphological similarity to *T. mentagrophytes/T. interdigitale* or other superficially similar species [21,27]. The emergence of *T. quinckeanum* in animals in recent years is also in concordance with the incidence of human cases (Figure 2).

Although we have no clear explanation for the sudden rise of *T. quinckeanum* infections substantiated by data, there are several possible hypotheses. The increase can be caused by an overpopulation of rodents in Czechia observed in previous years, potentially leading to an increased transmission to domestic animals and eventually humans [28,29]. This hypothesis, however, does not explain the geographic clustering of the majority of cases into the Olomouc region around Prostějov and Olomouc cities. All but four isolates came from this area, indicating a local outbreak. In addition, a study on the occurrence of dermatophytes in rodents was carried out in the Czech Republic in recent years, which did not detect the presence of this pathogen [30]. In contrast to previous sporadic infections due to *T. quinckeanum*, the spread of infections among domestic animals is probably the main driving force of the current outbreak similar to the German one [5]. The spread among domestic animals is a completely new phenomenon that has not been described and may be caused by a shift in the virulence and other biological properties of the pathogen.

Interestingly, the Czech and German strains show identical ITS rDNA sequences which were different from all other *T. quinckeanum* isolates in the GenBank database. This suggests that the current outbreaks were caused by genetically different strains with possible new features compared to *T. quinckeanum* strains occurring in the past. We hypothesize that these strains could be more virulent, easily transmissible from pets to humans, and able to cause a small epidemic. However, more detailed comparative studies mapping differences in the physiology, virulence and host preferences of current and historical strains are needed for conclusions to be drawn. In the last decade, several new or emerging pathogenic dermatophytes have been reported. Namely, *T. benhamiae* var. *benhamiae* is an emerging pathogen in Europe mostly transmitted to humans from guinea pig [19,31,32]. *Trichophyton erinacei*, a pathogen of hedgehogs, is increasingly reported from human infections worldwide due to the growing interest of people in pet hedgehogs [33,34,35]. Another member of the *T. benhamiae* complex, *T. persicum*, was discovered as one of the major causes of zoonotic infections in Iran [36]. Finally, the name *T. indotineae* (=*T. mentagrophytes* genotype VIII) was introduced for a presumed clonal offshoot of *T. mentagrophytes* which causes epidemic or tinea infections in India and some other countries [37]. In this respect, *T. quinckeanum* can be considered an emerging pathogen in Germany and the Czech Republic.

Uhrlaß et al. [5] recorded that the majority of patients were older than 50 years (40%). In contrast to the German study, the Czech patients were mostly in the age groups 19 to 49 years (48%) and 1 to 18 years (44%), with only two patients being older than 50 years. In agreement with Uhrlaß et al. [5], we observed higher infection rates in females. This can probably be explained by the fact that women more often cuddle their pets [5].

A recent study showed differences in specific parts of the mass spectrum between strains of *T. quinckeanum* and other common zoophilic dermatophytes such as the *T. mentagrophytes* and *T. benhamiae* [5], but it is not entirely certain that the MALDI Biotyper automatic identification mode is possible for *T. quinckeanum*. However, the specific peak in the 4050–4200 *m*/*z* spectrum for *T. quinckeanum* supports the possibility of routine differentiation from other zoophilic species in practice using the FlexAnalysis tool in combination with micro- and macromorphological features. Further comparisons are certainly required, mainly due to the intra-species variability of the *T. mentagrophytes/T. interdigitale* complex and similarities of mass spectra in other dermatophyte species, especially the closely related *T. schoenleinii*. As showed by Packeu et al. [38], one out of six strains of *T. quinckeanum* was incorrectly identified as *T. schoenleinii* using MALDI-TOF MS.

The standardized methodology E.Def 11.0 was recently updated by the EUCAST-AFST and reading of the 50% inhibition endpoint has been recommended [18]. Based on this approach, obtained results will be comparable in future studies, enabling the definition of the epidemiological cut-off values (ECOFFs) and breakpoints for the categorization of results. These days, ECOFF wild-type upper limits (WT-ULs) have been set for *T. rubrum* and *T. interdigitale* tested by the EUCAST E.Def 11.0 methodology for four antifungals (terbinafine, voriconazole, itraconazole and amorolfine). The proposed WT-ULs for terbinafine, itraconazole and amorolfine are 0.03, 0.25 and 0.125 for *T. rubrum* and 0.125, 0.25 and 0.5 for *T. interdigitale*, respectively [18]. Values obtained in all *T. quinckeanum* isolates were lower in comparison with both proposed cut-offs [18]. Articles on *T. quinckeanum* antifungal susceptibility testing are scarce. Niewerth et al. [39] tested one strain of *T. quinckeanum* for itraconazole (1 mg/L), terbinafine (0.001 mg/L) and ciclopirox (3 mg/L) and obtained comparable results to ours by using the broth microdilution test (results were read by eye as no visible growth).

According to the literature, *T. quinckeanum* infections were usually treated with various systemic and local antifungals, mainly their combinations. Griseofulvin, terbinafine, econazole, clotrimazole, bifonazole, ciclopirox and ketoconazole were successfully used in the therapy [5,6,22,25,40]. One case of bifonazole cream treatment failure has been described [25]. The affected animals in our study were successfully treated with *Pythium oligandrum* whole-body baths three times a week for 14 days, usually in combination with a disinfectant (Alfadin). This approach seems to be considerate to animals, well-tolerated and does not support the development of resistance. The activity of *P. oligandrum* against dermatophytes has been demonstrated in vitro and in a small sample of patients in vivo [41,42]. Although no large-scale clinical trials have been conducted, our study provides more evidence about the effectiveness of this treatment.

## 5. Conclusions

We described a second local outbreak of *T. quinckeanum* infections in addition to the recent outbreak in Germany. We also brought evidence that rising human infections were mostly mediated by infected cats and dogs among which the infection spread at least partly independent of rodents. The spread of these infections underscores the need for a closer collaboration between veterinarians, dermatologists, epidemiologists and public health personnel to set up appropriate preventive measures. The confirmation of species identification by molecular methods is desired because the pathogen morphologically resembles *T. mentagrophytes/T. interdigitale*, a species with a similar host spectrum. MALDI-TOF MS can help in distinguishing *T. quinckeanum* from other common zoophilic dermatophytes, especially in the MS range of 4110–4160 *m*/*z*. In vitro susceptibility testing showed that *T. quinckeanum* had low MICs for most antifungals tested, especially terbinafine. Elevated MIC values were recorded for fluconazole and ketoconazole.

## Figures and Tables

**Table 1:** Human isolates of *Trichophyton quinckeanum* examined in this study.

Sample ID	ITS Accession Number	Date of Isolation	Age	Place of Residence	Sex	Site of Lesion	Direct Microscopy	Contact with Animal	Treatment
CCF 5781	MZ312189	December 2016	23	České Budějovice	female	chin	negative	N/A	terbinafine, ciclopirox, clotrimazole
CCF 6513	MZ312190	November 2017	38	Měrovice	female	forearm	negative	cat, dog	N/A
CCF 6457	MZ312191	December 2017	77	Němčice nad Hanou	male	sole	positive	cat	N/A
CCF 6402	MZ312192	February 2018	47	Polkovice	female	calf, thigh, trunk	NP	N/A	terbinafine
CCF 6451	MZ312193	November 2018	13	Pardubice	female	back, shoulder	positive	cat	terbinafine
CCF 6403	MZ312194	November 2018	52	Vrbátky	female	trunk	NP	N/A	N/A
CCF 6514	MZ312195	November 2018	5	Přerov	female	scalp	negative	cat	N/A
CCF 6404	MZ312196	February 2019	9	Olomouc	male	chin	positive	cat, dog	N/A
CCF 6458	MZ312197	August 2019	5	Kojetín	male	scalp	negative	cat, dog	N/A
CCF 6405	MZ312198	September 2019	47	Olomouc	female	buttock	NP	cat	N/A
CCF 6406	MZ312199	September 2019	5	Čelechovice na Hané	female	scalp	negative	cat	terbinafine
CCF 6407	MZ312200	October 2019	44	Nezamyslice u Prostějova	female	shank	NP	dog, cattle	terbinafine
CCF 6459	MZ312201	December 2019	34	Hranice na Moravě	male	forearm	positive	cat, dog, rabbit	ciclopirox, terbinafine
CCF 6538; CCF 6453	MZ312202	December 2019	21	Prostějov	female	wound, pubic area	NP	N/A	terbinafine
CCF 6454	MZ312203	December 2019	41	Určice	female	trunk	NP	cat	terbinafine
CCF 6539	MZ312204	January 2020	15	Říčany	male	scalp	negative	N/A	N/A
CCF 6460	MZ312205	January 2020	38	Přerov	female	neck	positive	cat	ciclopirox
CCF 6455	MZ312206	January 2020	12	Náměšť na Hané	male	temporal area	NP	cat	N/A
CCF 6461; CLIS 1379/20	MZ312207	February 2020	31	Brno	female	thigh, forearm	positive	cat	N/A
CCF 6456	MZ312208	February 2020	9	Těšetice	female	thigh	NP	N/A	fusidic acid, ciclopirox, terbinafine
CLIS 6248	MZ312213	June 2020	30	Bučovice	female	forearm	negative	cat	N/A
CCF 6555	MZ312209	September 2020	30	Slavětín	male	trunk	positive	dog, rabbit, chinchilla	clotrimazole
CCF 6556	MZ312210	September 2020	8	Branky	female	thigh, chest	positive	cat	clotrimazole, ciclopirox
CCF 6589	MZ312211	September 2020	4	Dolní Lhota	female	thigh	positive	cat	clotrimazole, ciclopirox
CCF 6590	MZ312212	September 2020	5	Dolní Lhota	female	chin, knee, trunk, forearm	positive	cat	clotrimazole, ciclopirox

Legend: CCF—Culture Collection of Fungi, Department of Botany, Charles University, Prague, Czech Republic; NP—not performed; N/A—data not available.

**Table 2:** Animal isolates of *Trichophyton quinckeanum* examined in this study.

Sample ID	ITS Accession Number	Date of Isolation	Age	Animal Species, Race	Residence of the Owner	Sex	Site/Type of Lesion	Direct Microscopy	Treatment/ Outcome
CCF 6540	MZ312214	August 2019	3 m	European Shorthair cat	Prostějov	male	generalized annular lesions, particularly on the trunk and extremities	positive	Ecosin ^a^, the lesions healed after 14 days; the kitten was found on the street in Prostějov with another kitten (probably a sibling) also showing signs of dermatophytosis
CCF 6531	MZ312215	September 2019	7 y	European cat	Prostějov	male	the claw bed of the right truncal extremity; a crusty lesion sized approx. 0.5 cm	positive	Ecosin ^a^ and Alfadin; complete cure
CCF 6452	MZ312216	September 2019	2 m	European cat	Prostějov	female	the nose, neck, legs, inner sides of the paw pads; non-itchy crusty ^b^ lesions	positive	Ecosin ^a^ and Alfadin
CCF 6528	MZ312217	October 2019	5 m	Badger dog	Prostějov	female	the inner side of the right ear; a crusty lesion	positive	Alfadin, Ecosin ^a^ added; the lesion healed after 14 days
CCF 6515	MZ312218	November 2019	6 m	German Shepherd	Prostějov	male	nose sponge; a weeping crusty lesion	positive	amoxicillin-clavulanate, Ecosin ^a^, Alfadin; the lesion become smaller, probably healed, the owners did not come for the final check-up
CCF 6541	MZ312219	December 2019	3 m	European Cat	Prostějov	female	the nose and left ear; a crusty lesion	positive	Ecosin ^a^, Alfadin
CCF 6542	MZ312220	June 2020	2 m	European Cat	Prostějov	female	the entire body, particularly the paws	positive	Ecosin ^a^; several kittens from the same litter had signs of dermatophytosis
CCF 6529	MZ312221	June 2020	3 m	European Cat	Prostějov	male	scabs and crusts on the entire body, particularly the paws	positive	Ecosin ^a^
CCF 6530	MZ312222	July 2020	3 m	European Cat	Prostějov	female	a lesion above the right eye	positive	Ecosin ^a^
CCF 6554	MZ312223	October 2020	2 y 7 m	Dachshund	Prostějov	female	a lesion on the nose sponge	positive	Ecosin ^a^
CCF 6553	MZ312224	November 2020	5 y 3 m	American Bulldog	Prostějov	female	a weeping lesion on the lip	positive	cephalosporins without improvement, Ecosin ^a^ with shampoo containing miconazole and chlorhexidine, Alfadin

Legend: CCF—Culture Collection of Fungi, Department of Botany, Charles University, Prague, Czech Republic; m—months; y—years; ^a^ Ecosin (preparation containing *Pythium oligandrum*)—treatment by full body bath 3× a week for 14 days, if not stated otherwise; ^b^ suspected tinea infection in owners, probably transmitted from cat (results of mycological examination not available for this study).

**Table 3:** Physiological tests results for *Trichophyton quinckeanum* and comparison with selected *Trichophyton* species.

	Growth on Media ^a^								
Species (Number of Strains)	T1	T2	T3	T4	T5	T6	T7	Urease	Reference
*Trichophyton quinckeanum* (20)	+	+	+	+	+	-	+	+	this study
*T. benhamiae var. luteum* (20)	+	+	+	+	+	v	v	v	this study
*T. europaeum* (8)	+	+	+	+	+	+	+	+	this study
*T. erinacei* (18)	+	+	+	+	+	+	+	v	this study
*T. verrucosum* (NS)	-	+	+	+	v	-	-	v	[20,21]
*T. bullosum* (1)	-	-	+	+	-	-	-	+	this study
*T. mentagrophytes/T. interdigitale* (NS)	+	+	+	+	+	+	+	+	[21]

^a^ The tests were read after 7 and 14 days: T1—vitamin-free casamino acid agar, basal medium for agars T2–T5; T2—T1 + inositol; T3—T2 + thiamine; T4—T1 + thiamine; T5—T1 + nicotinic acid; T6—vitamin-free ammonium nitrate agar, basal medium for agar T7; T7—T6 + L-histidine; “-”—no growth; “+”—growth; “v”—variable (variable between strains of the same species); NS—the number of strains not specified.

**Table 4:** Antifungal susceptibility of 36 *Trichophyton quinckeanum* strains by EUCAST reference method E.Def 11.0.

Antifungal Agent	MICs by EUCAST (mg/L)				
	Reading Method	Range	GM	MIC50	MIC90
FLU	OD	2–64	18.38	16	64
	vis	16–>64	45.7	32	>64
TER	OD	0.004–0.016	0.004	0.004	0.004
	vis	0.004–0.08	0.005	0.004	0.004
ITR	OD	0.008–0.125	0.023	0.016	0.032
	vis	0.06–2	0.26	0.25	1
KET	OD	0.016–1	0.31	0.5	1
	vis	0.5–4	1.88	2	4
CLO	OD	0.008–0.125	0.037	0.032	0.064
	vis	0.125–0.5	0.26	0.25	0.5
AMO	OD	0.008–0.064	0.02	0.016	0.064
	vis	0.016–0.25	0.08	0.125	0.25
CIC	OD	0.008–1	0.21	0.25	0.5
	vis	0.25–1	0.67	1	1
EFI	OD	0.008–0.064	0.03	0.032	0.064
	vis	0.03–0.25	0.01	0.125	0.125

Antifungals: FLU—fluconazole; TER—terbinafine; ITR—itraconazole; KET—ketoconazole; CLO—clotrimazole; AMO—amorolfine; CIC—ciclopirox; EFI—efinaconazole; OD—optical density, i.e., MIC value read spectrophotometrically as 50% inhibition of the growth; vis—MIC value read visually as complete inhibition (no growth detected by eye); GM—geometric mean; MIC50 and MIC 90—minimum concentration at which 50% and 90% of the isolates were inhibited, respectively.

## Data Availability

The DNA sequences obtained in this study were deposited into the GenBank database (www.ncbi.nlm.nih.gov). The isolates were deposited into the Culture Collection of Fungi (CCF), Department of Botany, Charles University, Prague.

## References

[1] Dvořák J., Otčenášek M. (1969). Zoophilic dermatophytes commonly attacking man. Mycological Diagnosis of Animal Dermatophytoses.

[2] Ajello L., Bostick L., Cheng S.-L. (1968). The relationship of *Trichophyton quinckeanum* to *Trichophyton mentagrophytes*. Mycologia.

[3] Beguin H., Pyck N., Hendrickx M., Planard C., Stubbe D., Detandt M. (2012). The taxonomic status of *Trichophyton quinckeanum* and *T. interdigitale* revisited: A multigene phylogenetic approach. Med. Mycol..

[4] De Hoog G.S., Dukik K., Monod M., Packeu A., Stubbe D., Hendrickx M., Kupsch C., Stielow J.B., Freeke J., Göker M. (2017). Toward a novel multilocus phylogenetic taxonomy for the dermatophytes. Mycopathologia.

[5] Uhrlaß S., Schroedl W., Mehlhorn C., Krüger C., Hubka V., Maier T., Gräser Y., Paasch U., Nenoff P. (2018). Molecular epidemiology of *Trichophyton quinckeanum*—A zoophilic dermatophyte on the rise. J. Dtsch. Dermatol. Ges..

[6] Garcia-Sanchez M., Pereiro M., Pereiro M., Toribio J. (1997). Favus due to *Trichophyton mentagrophytes* var. *quinckeanum*. Dermatology.

[7] Hubka V., Čmoková A., Peano A., Větrovský T., Dobiáš R., Mallátová N., Lysková P., Mencl K., Janouškovcová H., Stará J. (2018). Zoonotic dermatophytoses: Clinical manifestation, diagnosis, etiology, treatment, epidemiological situation in the Czech Republic. Čes-Slov. Derm..

[8] Hubka V., Větrovský T., Dobiášová S., Skořepová M., Lysková P., Mencl K., Mallátová N., Janouškovcová H., Hanzlíčková J., Dobiáš R. (2014). Molecular epidemiology of dermatophytoses in the Czech Republic—Two-year-study results. Čes-Slov. Derm..

[9] Pihet M., Le Govic Y. (2017). Reappraisal of conventional diagnosis for dermatophytes. Mycopathologia.

[10] Kelly K.L. (1964). Inter-Society Color Council—National Bureau of Standards Color Name Charts Illustrated with Centroid Colors.

[11] Hubka V., Nissen C., Jensen R., Arendrup M., Cmokova A., Kubatova A., Skorepova M. (2015). Discovery of a sexual stage in *Trichophyton onychocola*, a presumed geophilic dermatophyte isolated from toenails of patients with a history of *T. rubrum* onychomycosis. Med. Mycol..

[12] Georg L.K., Camp L.B. (1957). Routine nutritional tests for the identification of dermatophytes. J. Bacteriol..

[13] Gräser Y., Kuijpers A.F.A., Presber W., De Hoog G.S. (1999). Molecular taxonomy of *Trichophyton mentagrophytes* and *T. tonsurans*. Med. Mycol..

[14] Gardes M., Bruns T.D. (1993). ITS primers with enhanced specificity for basidiomycetes-application to the identification of mycorrhizae and rusts. Mol. Ecol..

[15] White T.J., Bruns T., Lee S., Taylor J., Innis M.A., Gelfand D.H., Sninsky J.J., White T.J. (1990). Amplification and direct sequencing of fungal ribosomal RNA genes for phylogenetics. PCR Protocols: A Guide to Methods and Applications.

[16] Hubka V., Nováková A., Jurjević Ž., Sklenář F., Frisvad J.C., Houbraken J., Arendrup M.C., Jørgensen K.M., Siqueira J.P., Gené J. (2018). Polyphasic data support the splitting of *Aspergillus candidus* into two species; proposal of *Aspergillus dobrogensis* sp. nov.. Int. J. Syst. Evol. Microbiol..

[17] Sklenář F., Jurjević Ž., Houbraken J., Kolařík M., Arendrup M.C., Jørgensen K.M., Siqueira J.P.Z., Gené J., Yaguchi T., Ezekiel C.N. (2021). Re-examination of species limits in *Aspergillus* section *Flavipedes* using advanced species delimitation methods and proposal of four new species. Stud. Mycol..

[18] Arendrup M.C., Jørgensen K.M., Guinea J., Lagrou K., Chryssanthou E., Hayette M.-P., Barchiesi F., Lass-Flörl C., Hamal P., Dannaoui E. (2020). Multicentre validation of a EUCAST method for the antifungal susceptibility testing of microconidia-forming dermatophytes. J. Antimicrob. Chemother..

[19] Čmoková A., Kolařík M., Dobiáš R., Hoyer L.L., Janouškovcová H., Kano R., Kuklová I., Lysková P., Machová L., Maier T. (2020). Resolving the taxonomy of emerging zoonotic pathogens in the *Trichophyton benhamiae* complex. Fungal Divers..

[20] Lysková P., Hubka V., Petřičáková A., Dobiáš R., Čmoková A., Kolařík M. (2015). Equine dermatophytosis due to *Trichophyton bullosum*, a poorly known zoophilic dermatophyte masquerading as *T. verrucosum*. Mycopathologia.

[21] De Hoog G.S., Guarro J., Gené J., Figueras M.J. (2009). Atlas of Clinical Fungi.

[22] Besbes M., Cheikhrouhou F., Sellami H., Makni F., Bouassida S., Ayadi A. (2003). Favus due to *Trichophyton mentagrophytes* var. *quinckeanum*. Mycoses.

[23] Nenoff P., Herrmann J., Gräser Y. (2007). *Trichophyton mentagrophytes sive interdigitale*? A dermatophyte in the course of time. J. Dtsch. Dermatol. Ges..

[24] Hubálek Z., Kushwaha R.K.S., Guarro J. (2000). Keratinophilic fungi associated with free-living mammals and birds. Biology of Dermatophytes.

[25] Skořepová M., Štork J., Hrabakova J. (2002). Tinea gladiatorum due to *Trichophyton mentagrophytes*. Mycoses.

[26] La Touche C.J. (1959). Mouse favus due to *Trichophyton quinckeanum* (Zopf) MacLeod & Muende: A reappraisal in the light of recent investigations. Mycopathol. Mycol. Appl..

[27] Chollet A., Cattin V., Fratti M., Mignon B., Monod M. (2015). Which fungus originally was *Trichophyton mentagrophytes*? Historical review and illustration by a clinical case. Mycopathologia.

[28] Jacob J., Imholt C., Caminero-Saldaña C., Couval G., Giraudoux P., Herrero-Cófreces S., Horváth G., Luque-Larena J.J., Tkadlec E., Wymenga E. (2020). Europe-wide outbreaks of common voles in 2019. J. Pest Sci..

[29] Suchomel J., Šipoš J., Heroldová M. (2020). Gradace hraboše polního (*Microtus arvalis*) v roce 2019 v řepařských výrobních oblastech a její význam z hlediska škod na řepné produkci. Listy Cukrov. Řepař..

[30] Žárová Š. (2020). Dermatophytes Isolated from the Hair of Free-Living Rodents. Master’s Thesis.

[31] Drouot S., Mignon B., Fratti M., Roosje P., Monod M. (2009). Pets as the main source of two zoonotic species of the *Trichophyton mentagrophytes* complex in Switzerland, *Arthroderma vanbreuseghemii* and *Arthroderma benhamiae*. Vet. Dermatol..

[32] Nenoff P., Uhrlaß S., Krüger C., Erhard M., Hipler U.C., Seyfarth F., Herrmann J., Wetzig T., Schroedl W., Gräser Y. (2014). *Trichophyton* species von *Arthroderma benhamiae*—A new infectious agent in dermatology. J. Dtsch. Dermatol. Ges..

[33] Abarca M., Castellá G., Martorell J., Cabañes F. (2017). *Trichophyton erinacei* in pet hedgehogs in Spain: Occurrence and revision of its taxonomic status. Med. Mycol..

[34] Le Barzic C., Cmokova A., Denaes C., Arné P., Hubka V., Guillot J., Risco-Castillo V. (2021). Detection and control of dermatophytosis in wild European hedgehogs (*Erinaceus europaeus*) admitted to a french wildlife rehabilitation centre. J. Fungi.

[35] Hubka V., Peano A., Cmokova A., Guillot J., Seyedmousavi S., de Hoog G.S., Guillot J., Verweij P.E. (2018). Common and emerging dermatophytoses in animals: Well-known and new threats. Emerging and Epizootic Fungal Infections in Animals.

[36] Čmoková A., Rezaei-Matehkolaei A., Kuklová I., Kolařík M., Shamsizadeh F., Ansari S., Gharaghani M., Miňovká V., Najafzadeh M.J., Nouripour-Sisakht S. (2021). Discovery of new Trichophyton members, *T. persicum* and *T. spiraliforme* spp. November, as a cause of highly inflammatory tinea cases in Iran and Czechia. Microbiol. Spectr..

[37] Kano R., Kimura U., Kakurai M., Hiruma J., Kamata H., Suga Y., Harada K. (2020). *Trichophyton indotineae* sp. nov.: A new highly terbinafine-resistant anthropophilic dermatophyte species. Mycopathologia.

[38] Packeu A., Hendrickx M., Beguin H., Martiny D., Vandenberg O., Detandt M. (2013). Identification of the *Trichophyton mentagrophytes* complex species using MALDI-TOF mass spectrometry. Med. Mycol..

[39] Niewerth M., Splanemann V., Korting H.C., Ring J., Abeck D. (1998). Antimicrobial susceptibility testing of dermatophytes–comparison of the agar macrodilution and broth microdilution tests. Chemotherapy.

[40] Bilek J., Baranova Z., Kozak M., Fialkovicova M., Weissova T., Sesztakova E. (2005). *Trichophyton mentagrophytes* var. *quinckeanum* as a cause of zoophilic dermatomycosis in a human family. Bratisl. Lek. Listy.

[41] Gabrielová A., Mencl K., Suchánek M., Klimeš R., Hubka V., Kolařík M. (2018). The oomycete *Pythium oligandrum* can suppress and kill the causative agents of dermatophytoses. Mycopathologia.

[42] Načeradská M., Fridrichová M., Kellnerová D., Peková S., Lány P. (2017). Antifungal effects of the biological agent *Pythium oligandrum* observed in vitro. J. Feline Med. Surg..

